# Short-Range Berezinskii-Kosterlitz-Thouless Phase Characterization for the *q*-State Clock Model

**DOI:** 10.3390/e23081019

**Published:** 2021-08-07

**Authors:** Oscar A. Negrete, Patricio Vargas, Francisco J. Peña, Gonzalo Saravia, Eugenio E. Vogel

**Affiliations:** 1 Department of Physics, Universidad Técnica Federico Santa María, Vaparaíso 2390123, Chile; oscar.negrete@usm.cl (O.A.N.); francisco.penar@usm.cl (F.J.P.); 2 Center for the Development of Nanoscience and Nanotechnology, Santiago 8320000, Chile; eugenio.vogel@ufrontera.cl; 3 Department of Physics, Universidad de La Frontera, Casilla 54-D, Temuco 4811230, Chile; gonzalo.saravia@gmail.com

**Keywords:** *q*-state clock model, entropy, Berezinskii-Kosterlitz-Thouless transition, ergodicity

## Abstract

Beyond the usual ferromagnetic and paramagnetic phases present in spin systems, the usual *q*-state clock model presents an intermediate vortex state when the number of possible orientations *q* for the system is greater than or equal to 5. Such vortex states give rise to the Berezinskii-Kosterlitz-Thouless (BKT) phase present up to the XY model in the limit q→∞. Based on information theory, we present here an analysis of the classical order parameters plus new short-range parameters defined here. Thus, we show that even using the first nearest neighbors spin-spin correlations only, it is possible to distinguish the two transitions presented by this system for q greater than or equal to 5. Moreover, the appearance at relatively low temperature and disappearance of the BKT phase at a rather fix higher temperature is univocally determined by the short-range interactions recognized by the information content of classical and new parameters.

## 1. Introduction

The idea of using simple, discrete, and finite models to understand complex phenomena is a fundamental part of statistical physics. In particular, this guiding idea has achieved particular success in the study of continuous phase transitions. For an infinite system, in the critical regime, the correlation length diverges, and the system becomes scale-invariant. The critical phenomena can then be described employing field theory in the long-wavelength limit, and their physical properties are governed by universal critical exponents [1,2,3,4,5]. Two famous examples are continuous Landau Ginzburg-type phase transitions [6,7,8,9,10], which are driven by topological defects (vortices). A simple model that exhibits many of these fascinating features is the so-called *q*-state clock model, which is a discretized XY [11,12,13] spin model defined on the square lattice. We recently solved this model exactly for a very small system [14] and also on larger lattices up to square lattices 128 × 128 by Monte Carlo simulations, showing clearly the two-phase transitions and using two information theory approaches (mutability and diversity) and calculated in thermal equilibrium on the thermodynamic energy and magnetization variables as functions of temperature.

Moreover, the *q*-state clock model is one of many magnetic models to mimic the thermodynamics of some materials, and it can be viewed as a classical Heisenberg spins model with very strong planar anisotropy or the already mentioned discrete XY model. In its simplest form it consists of a system of N spins, $\vec{S_{i}}$ at site *i*, on a lattice where the spins can have *q* equivalent possible orientations or *“stations”* in two dimensions (2D), which can be written as ${\vec{S}}_{i}=\left(\sin\left(2\pi n_{i}/q\right),\cos\left(2\pi n_{i}/q\right)\right)$, where $n_{i}=0,1,2,\ldots ,q-1$. These spins are interacting with their nearest neighbors through an exchange energy *J*, so the Hamiltonian of this system can be written as follows.

(1) $$H=-\sum_{<i,j>}J\;\vec{S_{i}}\cdot \vec{S_{j}}\;,$$

where the sum runs over all pairs of nearest neighbors (i,j). If J>0 the system is ferromagnetic since the fundamental state is that all spins are aligned. The case q=2 corresponds to the well-known Ising model which will not be of much attention in the present paper.

A simulation in the canonical ensemble can be implemented to study properties as functions of temperature. One example is the magnetization that presents ferromagnetic ordering at low temperatures and paramagnetic disorder at very high temperatures. However, an intermediate phase arises for q≥5, giving rise to two-phase transitions [15,16,17,18,19,20,21]. The new ordering corresponds to the so-called Berezinskii-Kosterlitz-Thouless (BKT) phase where vortexes dominate ( BKT) [22,23]. Thus, the low critical temperature corresponds to the transition between a ferromagnetic phase (FP) to BKT, while the second transition at a higher critical temperature corresponds to the transition between the BKT phase to the usual disordered paramagnetic phase (PP). At values of q<5, this system only exhibits a single transition from an FP to a paramagnetic phase (PP). In this context, we highlight a particular work where through Monte Carlo simulations with nonconserved Glauber’s dynamics, the existence of two different transition temperatures for a finite *q*-state clock model with q≥5 is confirmed [24]. In that work, the transition temperatures are quantified using two different cumulants (the first transition temperature uses the Binder cumulant [25,26], while the second is characterized using a new cumulant defined by the authors).

The descent of the critical temperature for the FP to BKT phase transition is simple to understand: as *q* grows, less energy is needed to change the next spin to the next possible station. If two adjacent spins differ in orientation in δq=1 the extra energy per pair of spins is only $J2\pi^{2}/q^{2}$ which decreases as $q^{-2}$. So as *q* increases, the transition temperature FP to BKT decreases. The BKT phase at $T>T_{c1}$ is characterized by wave-like spin excitations and vortexes since their energies are very close to that of the ground state. Therefore when q→∞ the FP to BKT transition temperature $T_{c1}\to 0$. The characterization of the different phases can be achieved by calculating the specific heat, or the 2D order parameter, which is the spin thermal average in the *X* and *Y* directions [14,27]. One of the main purposes of this work is to show that both phase transitions can also be characterized by appropriate short-range order parameters defined below, using simple spin correlations up to second and third nearest neighbors.

In the present paper, we want to get deeper into the vorticity reached by the BKT phase by two different means: On the one hand, we fully invoke diversity as the most sensitive tool provided by information theory to achieve this goal (mutability and Shannon entropy agree with these results but produce less sharp transition curves). On the other hand, we will define new and more appropriate parameters to recognize the way vortexes appear and disappear, establishing the way their presence marks both transitions.

This article is organized in the following way: Next Section describes the system. Section 3 covers the methodology from different points of view, including the definition of the new short-range order parameters. Section 4 is devoted to the presentation of the new results and their discussion. Section 5 includes the main conclusions of this paper.

## 2. System

**Figure 1:**
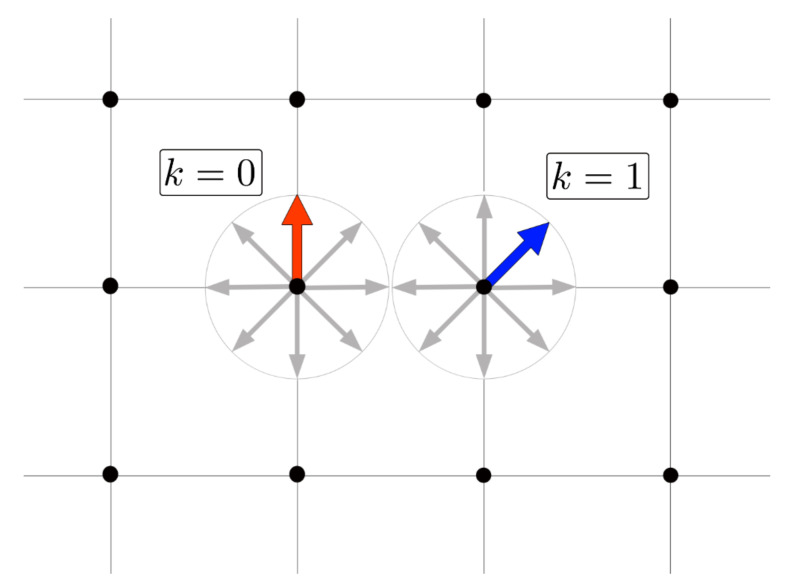
Two neighboring spins with orientations k=0 and k=1 interacting on a square lattice for *q* = 8.

Let us consider a square lattice L×L=N with one magnetic unit or spin at each site *i*. These spins lay on the plane of the lattice and have *q* fixed possible orientations or stations at angles kπ/q with k=0,1,2,…,q−1 (see Figure 1).

The Hamiltonian defining this interaction is given by Equation (1) where we express energy in units of *J* (J=1 unit of energy); temperature can also be expressed in the same scale (Boltzmann constant is dimensionless and equal the unity).

The lattice average of the spin configuration, equivalent to the magnetization per site *m*, is given by the following expression:

$$\vec{m}=\frac{1}{N}\sum_{j=1}^{N}{\vec{S}}_{j}$$

where $S_{j}=\left(\sin\left(2\pi n_{j}/q\right),\cos\left(2\pi n_{j}/q\right)\right)$ with $0\leq n_{j}\leq q-1$ is the value of the spin at site *j* at a given time *t*, and N=L×L is the total number of spins. In this particular case, $\vec{m}$ is a vector of two components, $\vec{m}=\left(m_{x},m_{y}\right).$

The normalized absolute magnetization is defined by the following relation.

(2) $$<|\vec{m}|>=\frac{1}{N_{c}}\sum_{k=1}^{N_{c}}\sqrt{m_{kx}^{2}+m_{ky}^{2}}$$

where $N_{c}$ is the number of configurations used to perform thermal averages for state properties.

This form of looking at the magnetization will recognize long-range magnetic correlations that include the BKT phase. However, if we want to detect the ergodicity breaking associated with the short-range ordering, we have to look at the magnetization along the natural *q* directions of the system. Let us define $m_{k}$ as the normalized magnetization along the *k*-th direction only, namely, it presents the fraction of spins pointing along the *k*-th direction. If we start from a very low temperature, then the phase will be ferromagnetic along just one of these directions, κ say, while the magnetization along the oher directions vanish. Namely, in the limit T→0, $m_{k}=1.0$ for k=κ, while $m_{k}=0.0$ otherwise. We say, κ is the dominant direction.

As *T* first increases, some spins deviate from the κ direction, and $m_{\kappa }$ slightly weakens in favor of other directions. As *T* continues to increase, the *dominant* direction may shift to another different from κ, and the spontaneous magnetization direction of the system will be different. Thus, for instance, if the system was floating in the presence of an external weak magnetic field, it will now realign its direction according to its new magnetization axis. Let us define the *dominant magnetization axis D* at a given temperature *T* as the one that presents the highest fraction of spins pointing along the direction defined by this axis. Namely, among all the *q* directions at the time of observation, $m_{D}\left(T\right)$ represents the largest number of spins pointing in the direction k=D at a given temperature.

## 3. Methodology

### 3.1. Calculations and Data Organization

All calculations are done for time series generated by Monte Carlo simulations on a square lattices of N=L×L sites; a standard Metropolis algorithm was used. Global properties like energy, specific heat and magnetization are faster and were done for L=128 with 120,000 MC steps for equilibration plus additional 120,000 MC steps to generate the average value for each temperature (one MC step means L×L spin-flips attempts).

State-oriented properties, such as spin-spin correlations, are slower and were done by employing $5\times 10^{4}$ MCS for equilibrium, additional $10^{4}$ MCS for measurements at intervals of 50 MCS are stored in a matrix file. These values will be later used to calculate average values, standard deviations, and the information recognition for the classical variables. In addition, short range spin-spin correlation defined below will also be calculated, requiring the storage of all spin orientations at any given instant. Such a task is slow and memory consuming which imposes a limit to the present calculations. So we restrict ourselves to q=2,…,8, L=32, and 64, and with a Monte Carlo sequence initiated at low temperatures up to 3.0 in terms of *J*, with a ferromagnetic initial configuration.

### 3.2. Information Recognizer

Data compressor wlzip was created to recognize repeated meaningful information in any data sequence [28,29,30,31,32,33,34,35,36,37]. It is offered free of charge upon request by email (eugenio.vogel@ufrontera.cl). Actually, wlzip is less efficient than other compressors in terms of the final size achieved by the compressed file. However, compression done by wlzip is based on exact matching of data structures that correspond to properties of the system [28,29,30,31,32,33,34,35,36,37]. A high degree of compression is due to repetitive information characterizing a system that tends to preserve its properties within the time window under consideration. A very low degree of compression means that the system changes frequently and/or abruptly the properties represented by the data in the file; a chaotic system will be among the less compressible cases.

These considerations lead us to the definition of the information theory functions used here. Let us consider a vector file with ν entries for any of the possible variables as those generated by the MC calculations described above. Let W(ν) denote the size of this file in bytes. Then an appropriate compressor (like wlzip) is invoked to generate a compressed file of size $W^{*}\left(\nu \right)$; then the mutability μ(ν) for this variable, for a sequence of ν entries, at the conditions this vector file was generated is given by:

(3) $$\mu \left(\nu \right)=\frac{W^{*}\left(\nu \right)}{W(\nu )}\;.$$

Wlzip generates something similar to a histogram where each different exact value of the property defines a bean (within numeric precision that can be externally adjusted). There is a difference thought: the entries in the compressed files keep track of their relative positions in the original file thus providing dynamic information. Let $\lambda^{*}\left(\nu \right)$ be the number of beans (different number of values of these properties among ν entries) then the diversity div(ν) is defined as:

(4) $$div\left(\nu \right)=\frac{\lambda^{*}\left(\nu \right)}{\nu }\;.$$

If the dynamic information is ignored and the beans are treated as those of a normal histogram then the number of entries $n_{j}$ with value $f_{j}$ within the compressed files allows us to define the probability $p_{j}\left(\nu \right)$ of visiting this *j*-th state within this time series of ν values as

(5) $$p_{j}\left(\nu \right)=\frac{n_{j}}{\nu }\;.$$

With this state probability we can calculate the Shannon entropy by its usual way:

(6) $$h\left(\nu \right)=-\sum_{j}p_{j}\left(\nu \right)ln\left(p_{j}\left(\nu \right)\right)\;.$$

Information recognition is done on the vector files storing decimal information with four active digits and no truncation in the data recognition is performed. This feature can be improved if a precise determination of critical temperatures is needed [31]. Since in the present paper the main goals are methodological, we do not proceed further in the refinement of the data recognition.

### 3.3. Short-Range Order Parameters

**Figure 2:**
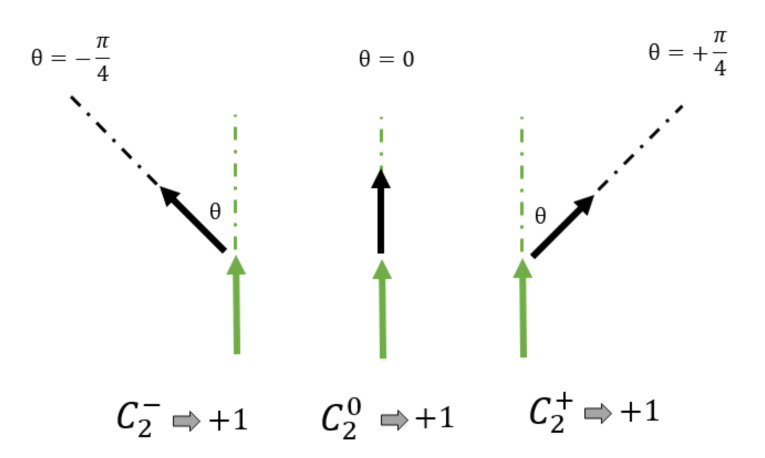
Graphical definition of two-spin parameters.

**Figure 3:**
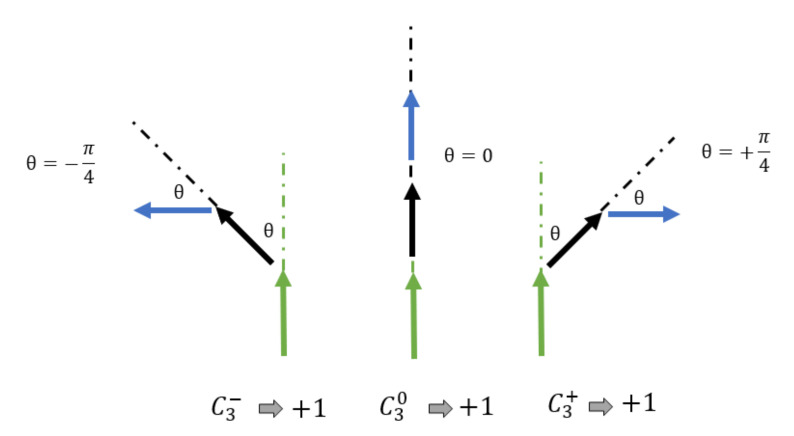
Graphical definition of three-spin parameters.

We define short-order parameters to recognize the surge and disappearance of vortexes in the system. Two-spin order parameter $C_{2}^{0}$ is intended to recognize that two neighboring spins possess the same orientation regardless of the *k*-th orientation among the *q* orientations of the system. $C_{2}^{+}$ ($C_{2}^{-}$) recognizes when the second spin along the direction of the spin of the former deviates from the first one in an angle θ=+π/4 (θ=−π/4) clockwise (counterclockwise). This is illustrated in Figure 2. Three-spins parameters are defined in a similar way as illustrated in Figure 3; first and second nearest neighbors are considered. $C_{3}^{0}$ intends to recognize three consecutive spins pointing along any of the *q* directions of the system, while $C_{3}^{+}$ and $C_{3}^{-}$ recognize the continuation of the curling of the first pair through the third spin. Thus we go over the entire lattice, counting each couple or trio just once, adding unity to the corresponding parameter, and normalizing over the *N* number of different sets.

The procedure illustrated above is thought for a q=8 system, but it can be modified and adapted to other systems: (i) Let us assume that calculations have stopped and we go through the lattice with the last state visited as a reference; (ii) We go to sites sequentially; (iii) Consider site *i* and look in the direction that points the spin $\vec{S_{i}}$ to find next spin along this direction; (iv) if that spin points in the same direction as $\vec{S_{i}}$, θ=0 and $C_{2}^{0}$ is incremented; if that spin points in a direction that deviated in an angle +2π/q$\left(-2\pi /q\right)$ with respect to $\vec{S_{i}}$ then $C_{2}^{+}$ ($C_{2}^{-}$) is incremented. Namely, a still not normalized counter increases:

(7) $$C_{2}^{0}\to C_{2}^{0}+1$$

Once the counting procedure finishes the counter is normalized to its final form:

(8) $$C_{2}^{0}\to \frac{C_{2}^{0}}{N}$$

and similarly for $C_{2}^{+}$ and $C_{2}^{-}$.

The definition of the three-spin parameters goes along the same way, requiring the same conditions along three consecutive spins and it is illustrated in Figure 3.

It is convenient to define average values for the curling parameters in the form:

(9) $$C_{2}^{A}=\left(C_{2}^{+}+C_{2}^{-}\right)/2;\;C_{3}^{A}=\left(C_{3}^{+}+C3^{-}\right)/2\;.$$

At the ferromagnetic initial state at very low temperature, it is obvious that $C_{2}^{0}$ as well as $C_{3}^{0}$ yield both 1.0, the same result of the normalized magnetization.

## 4. Results and Discussion

The internal energy of the system can be obtained from MC simulations, as depicted in the methodology presented above. Then, its temperature derivative leads us to the specific heat C(T), whose results for different *q* values can be appreciated, for instance, Ref. [14]. A clear maximum is observed in the C(T) curves for q≤4, changing to a maximum and a shoulder for q≥5. A phase diagram presented in the same previous reference clarifies that this can be thought of as one phase transition originating in the loss of the FP to a PP for q≤4 and to a BKT phase for q≥5; the critical temperature associated to this phase transition decreases monotonously towards zero as q→∞. The second transition arises from the loss of the BKT into a PP, and its critical temperature is rather independent of *q* at nearly 1.1. Such results are also confirmed by the changes in magnetization for the same MC simulations.

**Figure 4:**
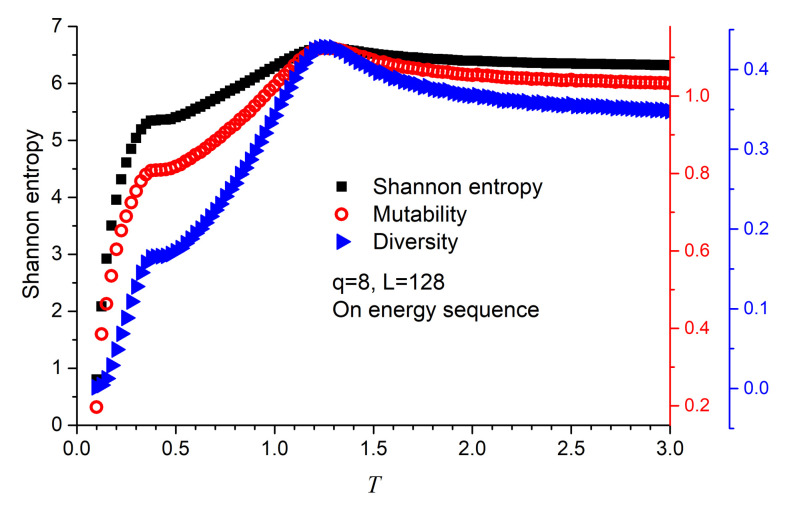
Information theory parameters as functions of temperature for q=8 and L = 128. Each point corresponds to a measurement done on the energy sequence of 120,000 instants after equilibrium. The scales for the three dimensionless parameters have been adapted to begin as similar values, coinciding in the maxima, stretching the same span for comparison purposes. The most external ordinate axis to the right corresponds to diversity.

These previous results can also be obtained through information theory which is now complemented by including Shannon entropy as shown in Figure 4 for q=8 in a lattice with L=128 (no significant differences are obtained for other *L* values). It is not surprising that these three curves (as well as specific heat or magnetic susceptibility) maximize at nearly the same temperature. It is clear that any sequence of data representing an observable of a system will be altered near the critical temperatures. The recognition of this alteration can be obtained in different ways. In the present case, if a count of frequencies is obtained for the different values visited when measuring the magnetization we would obtain a distribution. Suppose a magnetization histogram is constructed with this information; then the normalized visits to any of these values will allow to sample the probability of visiting that value; this leads directly to the Shannon entropy according to Equation (6); the span of the values visited will lead to the diversity (given by Equation (4)); the relative size of the compressed file with respect to the original file containig the magnetization series will yield the mutability. It should be noticed that Shannon entropy and diversity pay no attention to time while mutability bears an indirect reference to time [29,34]: the sooner a value repeats itself in the series the lighter the compressed file results.

**Figure 5:**
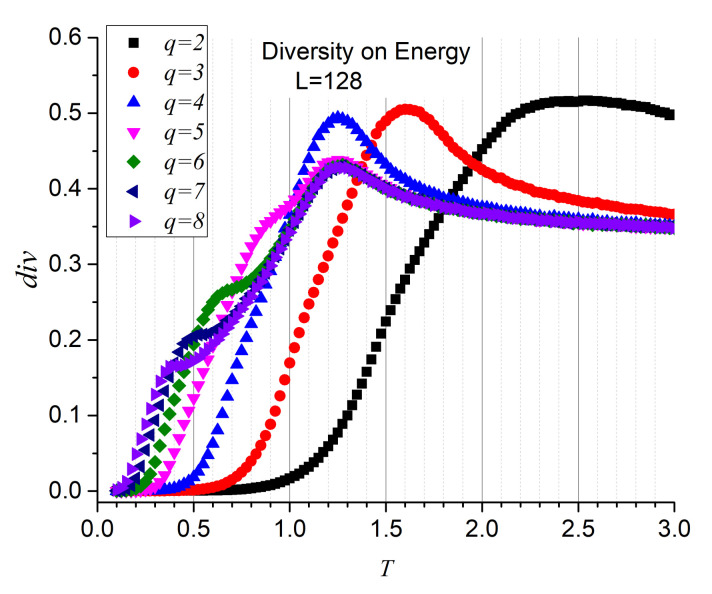
Diversity for energy series of 120,000 instants as functions of temperature for *q* = 2, …, 8, on an L=128 lattice.

**Figure 6:**
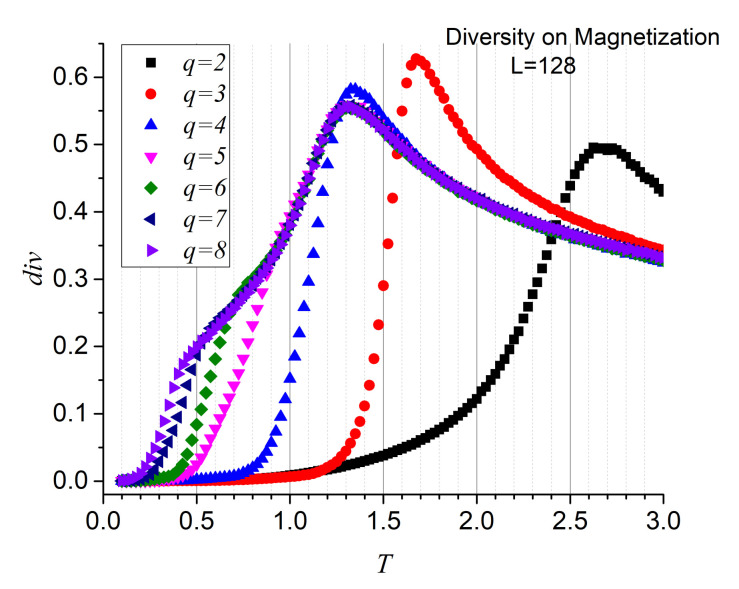
Diversity for magnetization series of 120,000 instants as functions of temperature for *q* = 2, …, 8, on an L=128 lattice.

Although the three functions maximize at the precise temperatures, it is the diversity that reports the second maximum more sharply. For this reason, we stick to diversity only from now on. Figure 5 render the diversity results for the energy series for q=2,3,…,8, confirming both transitions and values of the critical temperatures. Additionally, Figure 6 reports the diversity results for the magnetization series, confirming that the origin of these transitions is of magnetic nature. These are significant results since they fully incorporate information analysis as a tool for recognizing the FP- BKT-PP transitions.

In spite of the recognition of the transition phases, there are no reports on the exact nature by which the BKT phase appears, evolves, and later disappears with temperature. It is only the fluctuations of energy and magnetization that reveals the change of state as a gross feature. We now turn our attention to the new parameters defined by Equation (7) and Figure 2 and Figure 3 to better characterize the short-range order implied by the BKT phase. When applicable, we will prefer the case q=8 to illustrate the vortex state since it is a system where the BKT phase is clearly present and the eight orientations for the clock model are rather intuitive at π/4 angles between them.

**Figure 7:**
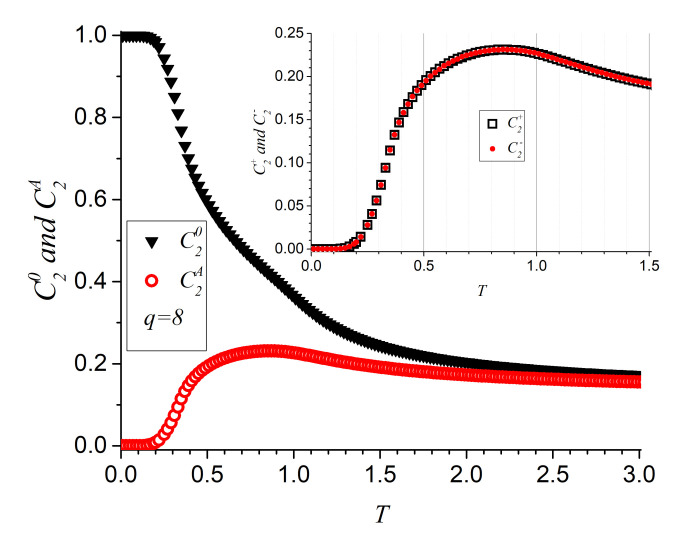
Two-spin parameters as functions of temperature for q=8; L=64 and after 10,000 MC steps post equilibrium. The main body plot $C_{2}^{0}$ and $C_{2}^{A}$, while the inset plots $C_{2}^{+}$ and $C_{2}^{-}$ separately.

Figure 7 presents the variations of $C_{2}$ parameters as functions of temperature; a change in the curvature of the decaying magnetization in the form of a slight “swelling” can be appreciated. The lower curve with open symbols gives the average value $C_{2}^{A}$ defined in Equation (9) that presents a broad maximum at T≈0.8 (the same temperature at which $C_{2}^{0}$ presents the commented swelling). The inset presents separate results for $(C_{2}^{-}$ and $C_{2}^{+}$ showing that they give almost identical results (as they should since there is no anisotropy). The interpretation of these curves is direct: the system is initiated as ferromagnetic, so $(C_{2}^{-}$ and $C_{2}^{+}$ are both zero; slightly over T=0.2 some spins randomly get enough energy to orient their magnetization to the next station at either side (π/4 or −π/4) and parameters $(C_{2}^{-}$ and $C_{2}^{+}$ begin to grow; the growth reaches maximum pace at around T=0.4, and the abundance of these parameters tends to maximize near T=0.8. From there on, energy is high enough, so excitations to larger angles are also possible, and the two-spin vortex parameters slowly decrease to their asymptotic limit for huge temperatures, which is typical for two-spin parameters at any angle, namely 1/q (0.125 in the scale of Figure 7).

**Figure 8:**
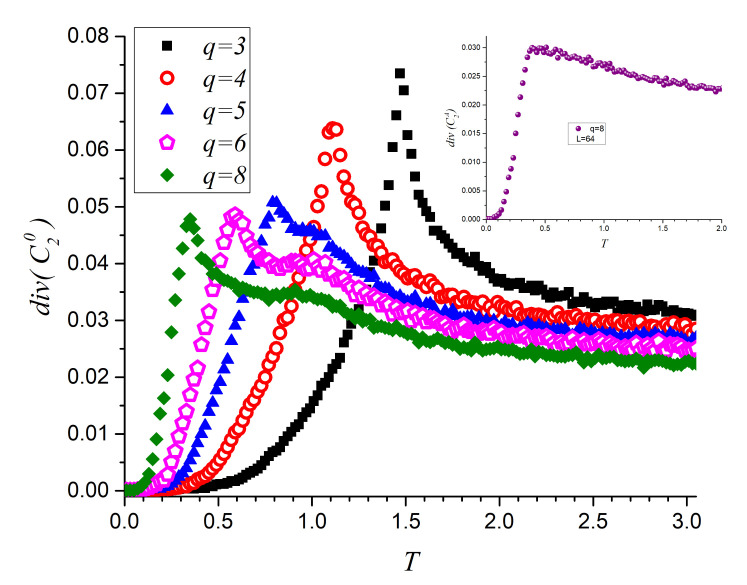
Diversity of $C_{2}^{0}$ for q=3,4,5,6 and 8. ($C_{2}^{7}$ is omitted for clarity). The inset presents the diversity of the average $C_{2}^{A}$ parameter.

The information content in the $C_{2}$ series is reported through the diversity of $C_{2}^{0}$ in Figure 8 for q=3, 4, 5, 6 and 8 which confirm previous findings by this additional method. Namely, for q=3 just one peak is found just above T=1.5; this maximum decreases to T=1.1 for q=4, while for q=5, q=6 and q=8 the low temperature peak keeps on moving to lower temperatures while the high temperature peak sticks around T≈1. All of this in correspondence with previous gross feature results (Figure 5 in particular). The inset of Figure 8 reports on the average diversity of the curling two-spin parameters ($[C_{A}=C_{2}^{+}+C_{2}^{-})/2]$) that maximizes nearly at T=0.4 in agreement with the greatest slope in the inset of Figure 7.

**Figure 9:**
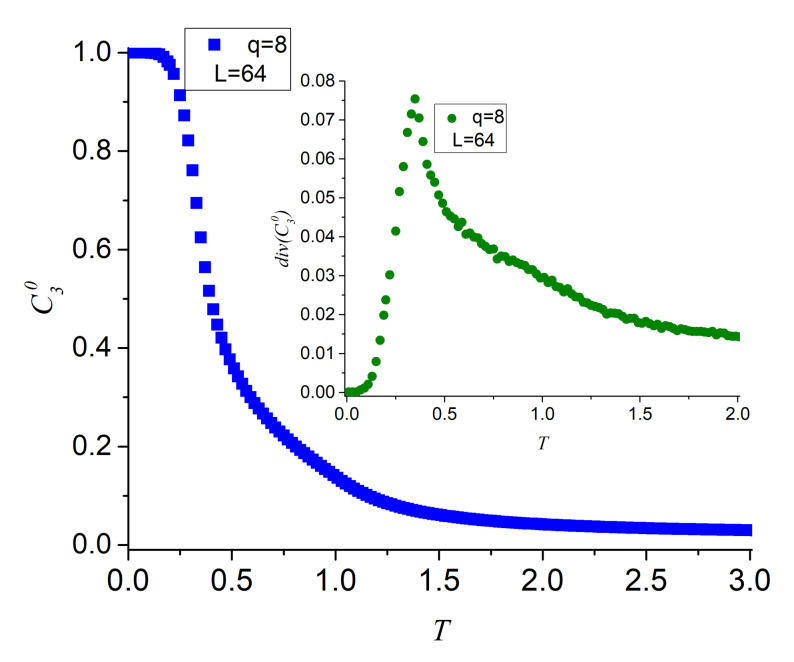
Three-spin parameter $C_{3}^{0}$ descending from a full ferromagnetic state to its asymptotic limit ($1/q^{2}$) with a slight swelling under T=1.0 as a function of temperature for q = 8. The inset presents the diversity of this parameter maximizing near 0.4 (as $C_{2}$ did) and a slight swelling near 1.0.

Figure 9 presents the descent of $C_{3}^{0}$ parameter as functions of temperature in a way similar to $C_{2}^{0}$. The inset presents the diversity of $C_{3}^{0}$ that maximizes at the point of the more pronounced descent of the parameter itself (main figure). Parameters $C_{3}^{+}$ and $C_{3}^{-}$ (not shown) behave similarly to the corresponding two-spin parameters $C_{2}^{+}$ and $C_{2}^{-}$, except that their curves maximize as they approach T=1.0 since spins need to be freer by effect of temperature to articulate a series of two consecutive π/4 (−π/4) angles. The inset reports the diversity of $C_{3}^{0}$ maximizing at the same temperature $C_{2}^{0}$ did in the inset of Figure 7 evidencing that they both represent ferromagnetic alignment. The inset of Figure 9 presents an additional disctintive feature: a slight broad swelling near T=1.0 can be seen for $div(C_{3}^{0})$ evidencing the disappearance of three-spin correlations; this effect will appear again in a different way after the next paragraph.

**Figure 10:**
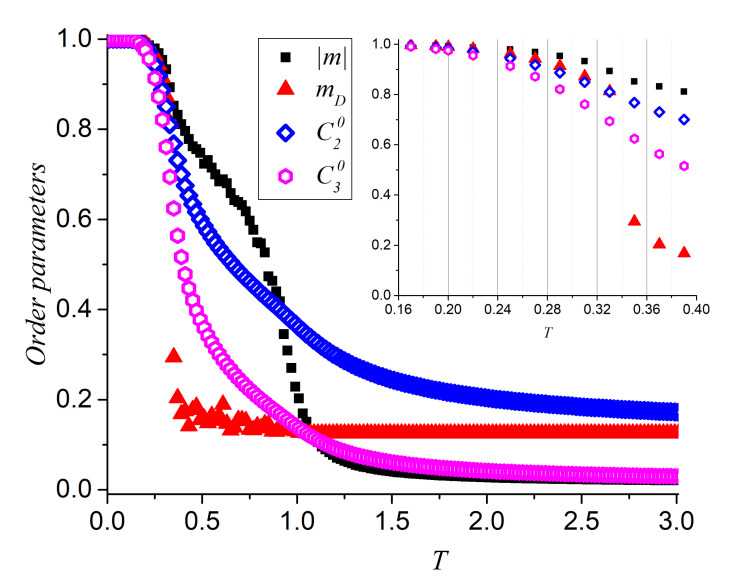
Order parameters $C_{2}^{0}$, $C_{3}^{0}$ compared to the dominant magnetization $m_{D}$ (along any of the 8 directions) and the absolute value of the normalized magnetization |m| for q=8. In the low temperature range (inset), we show the decrease of the ferromagnetic spin-spin correlation with temperature that occurs first for $C_{3}^{0}$, then for $C_{2}^{0}$, followed by $m_{D}$ and closing with the absolute magnetization.

The descents of $C_{2}^{0}$ and $C_{3}^{0}$ resemble a bit like the magnetization curves for these systems. In Figure 10 we do this comparison for q=8. The four parameters presented in this figure measure similar properties but with subtle differences: $C_{2}^{0}$ measures the way in which the pairing of two parallel spins is gradually lost tending to its asymptotic value 1/8; $C_{3}^{0}$ measures the way in which the pairing of three parallel spins is lost at lower temperatures than previous case tending to its asymptotic value of 1/64 (first spin is a pivot and second and third spins have independents probabilities 1/8 to point along the curling direction). The absolute magnetization curve given by Equation (2) changes twice its slope evidencing the two changes of phase and tending asymptotically to zero. On the other hand, $m_{D}$ measures the way the magnetization loses its dominant direction originated in the spontaneous ergodicity breaking associated to the FP- BKT phase transition only, as defined at the end of Section 2. Thus, this figure offers a complete picture of the magnetization evolution of the system as *T* increases: at low-temperature, ergodicity is broken in favor of a ferromagnetic ordering along one dominant direction; then the FP is lost as drastically shown by the abrupt descent of $m_{D}$; at T increases the absolute magnetization, $C_{2}^{0}$ and $C_{3}^{0}$ point to the presence of short magnetic ordering, with ergodicity recovering with the increase of *T*; At a temperature in which the slightest short-range interaction is exceeded by the thermal fluctuations (this critical temperature is unique) the PP is finally reached and ergodicity is fully recovered. We observe in the low temperature range that the decrease of the ferromagnetic spin-spin correlation occurs first for $C_{3}^{0}$, then $C_{2}^{0}$ and finally the extended order parameters, i.e., the dominant magnetization and the absolute magnetization.

**Figure 11:**
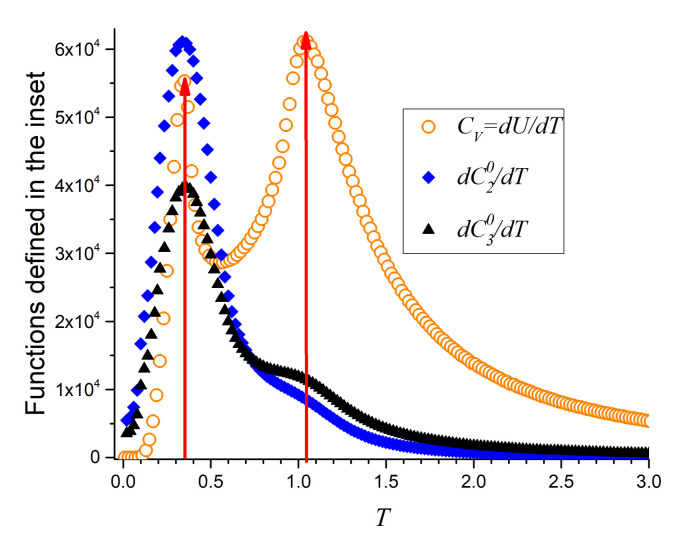
Temperature derivatives of variables *E*, $C_{2}^{0}$ and $C_{3}^{0}$ coinciding in point to both phase transitions for q=8 as an example.

If careful attention is paid to Figure 7 and Figure 9 it can be noticed that $C_{2}^{0}$ and $C_{3}^{0}$ present changes of curvature. This is better analyzed by taking the temperature derivatives of these curves which are presented in Figure 11, where they are compared to the specific heat of the same particular system (q=8). It is clear that the derivatives of the spin parameters showing ferromagnetic alignments maximize at both transitions thus providing an additional argument and measurements to define these magnetic transitions. The low temperature maximum is more pronounced and agrees perfectly with the corresponding maximum of the specific heat. In the case of the highest transition temperature, the temperature derivative of $C_{3}^{0}$ shows a larger broad maximum than the temperature derivative of $C_{2}^{0}$ in consonance with the light swelling shown by the diversity of $C_{3}^{0}$ in the inset of Figure 9.

## 5. Conclusions

Information content of the series corresponding to the classical variables internal energy and magnetization recognize the different phase transitions present in the clock model. Among the three investigated information theory techniques (Shannon entropy, mutability, and diversity), it is diversity that provides the sharpest curves with appropriate contrast to better recognize the transitions.

The new parameters defined here following the curling lines of the vortexes give information on the short-range ordering achieved by the system. Thus, the ferromagnetic phase is first lost to two-spin vortex parameters at π/4 just under T=0.4, which is then complemented by the less frequent three-spin parameters with two consecutive twists of π/4. The complete BKT phase receives contributions from all possible *n*-spin vortex parameters rendering a composed critical temperature around T=1.1 for the BKT-PP transition.

Diversity of the time series confirms both transitions for q≥5 and just one transition for q≤4 in perfect agreement with the phase transitions already reported by reference [14].

$C_{3}$ parameters are more sensitive than $C_{2}$ parameters since the former actually measure the formation of three-spin vortexes, while the former arise from the loss of the original ferromagnetic phase.

All *q*-clock systems have a similar critical temperature for the BKT-PP phase transition since this is mainly due to the dissociation of correlation in the interaction of neighboring spins, thus affecting the $C_{2}^{+}$ and $C_{2}^{-}$ parameters, which are at the bases of more complex *n*-spin parameters.

Temperature derivatives of the new parameters are in perfect agreement with the temperature derivative of the internal energy (specific heat) further validating the new parameters as describing the phenomenon which can now be also explained in terms of short-range ordering. Finally, we can conclude that the spin-spin ferromagnetic correlation at first-nearest neighbors is able to recognize the two phase transitions, FP to BKT and BKT to PP, confirming the same transition temperatures of the *q*-state clock model for q≥5.
